# Multi-gesture drag-and-drop decoding in a 2D iBCI control task

**DOI:** 10.1088/1741-2552/adb180

**Published:** 2025-04-10

**Authors:** Jacob T Gusman, Tommy Hosman, Rekha Crawford, Tyler Singer-Clark, Anastasia Kapitonava, Jessica N Kelemen, Nick Hahn, Jaimie M Henderson, Leigh R Hochberg, John D Simeral, Carlos E Vargas-Irwin

**Affiliations:** 1 Biomedical Engineering Graduate Program, School of Engineering, Brown University, Providence, RI, United States of America; 2 School of Engineering, Brown University, Providence, RI, United States of America; 3 Robert J. and Nancy D. Carney Institute for Brain Science, Brown University, Providence, RI, United States of America; 4 VA Center for Neurorestoration and Neurotechnology, Office of Research and Development, VA Providence Healthcare System, Providence, RI, United States of America; 5 Department of Neuroscience, Brown University, Providence, RI, United States of America; 6 Center for Neurotechnology and Neurorecovery, Department of Neurology, Massachusetts General Hospital, Boston, MA, United States of America; 7 Department of Neurosurgery, Stanford University, Stanford, CA, United States of America; 8 Wu Tsai Neurosciences Institute, Stanford University, Stanford, CA, United States of America; 9 Bio-X Program, Stanford University, Stanford, CA, United States of America; 10 Department of Neurology, Harvard Medical School, Boston, MA, United States of America

**Keywords:** brain–computer interfaces, human computer interaction, human motor cortex, paralysis, spinal cord injury

## Abstract

*Objective*. Intracortical brain–computer interfaces (iBCIs) have demonstrated the ability to enable point and click as well as reach and grasp control for people with tetraplegia. However, few studies have investigated iBCIs during long-duration discrete movements that would enable common computer interactions such as ‘click-and-hold’ or ‘drag-and-drop’. *Approach*. Here, we examined the performance of multi-class and binary (attempt/no-attempt) classification of neural activity in the left precentral gyrus of two BrainGate2 clinical trial participants performing hand gestures for 1, 2, and 4 s in duration. We then designed a novel ‘latch decoder’ that utilizes parallel multi-class and binary decoding processes and evaluated its performance on data from isolated sustained gesture attempts and a multi-gesture drag-and-drop task. *Main results*. Neural activity during sustained gestures revealed a marked decrease in the discriminability of hand gestures sustained beyond 1 s. Compared to standard direct decoding methods, the Latch decoder demonstrated substantial improvement in decoding accuracy for gestures performed independently or in conjunction with simultaneous 2D cursor control. *Significance*. This work highlights the unique neurophysiologic response patterns of sustained gesture attempts in human motor cortex and demonstrates a promising decoding approach that could enable individuals with tetraplegia to intuitively control a wider range of consumer electronics using an iBCI.

## 1. Introduction

Intracortical brain–computer interfaces (iBCIs) utilize neural signals from motor areas of the brain to allow people with tetraplegia to control external devices like computers and robots using imagined or attempted movements of the arm and hand [1–4]. Recent work has shown that individual finger movements [5], complex hand gestures [6], and even handwriting [7] can be reliably decoded using iBCIs. However, each of these control signals represent transient motor commands that evolve over the course of 1 or 2 s and are decoded as discrete events. Many upper extremity actions important to everyday activities require maintaining a specific posture over relatively longer periods of time (holding objects, pressing a button for a desired duration, clicking and dragging with a computer mouse, etc). How do cortical signals evolve during this type of sustained, static motor command?

Neurons in motor and premotor cortices of nonhuman primates (NHPs) have frequently been characterized as ‘phasic-tonic’; neurons in these areas tend to fire more robustly at the beginning of an isometric grasp attempt, but less so as the grasp force is maintained over time [8, 9]. While neural activity during initial phasic responses tends to correlate with EMG, sustained tonic activity is only weakly correlated with motor output [9–11]. Furthermore, the tuning of NHP motor cortical neurons to different motor commands can decrease over the course of a sustained isometric hold [12, 13], which would make it more difficult for a neural decoder to reliably differentiate between different sustained actions held over time. Additionally, grasp decoding of sustained holds can be attenuated during concurrent arm translation [14].

To date, the majority of iBCI neural decoding systems circumvent these challenges by employing velocity-based control logic. Attempted movements performed by the participant are mapped to discrete or continuous changes in the state or position of cursors and robotic effectors [2, 15–19]. Recent work has shown that intuitive drag-and-drop control of a cursor could be approximated by detecting transient signals corresponding to the initiation and release of a single hand grasp [20]. Here, we utilize a neural ‘latch’ decoder to accommodate sustained gesture attempts performed in a multi-gesture context. Through this approach, we demonstrate the possibility of offering responsive and consistent decoding of multiple gestures over long hold periods.

## 2. Methods

### 2.1. Participants

Study participants T11 and T5 provided informed consent and were enrolled in the pilot clinical trial of the BrainGate Neural Interface System. Enrollment criteria and details about the trial can be found at http://www.clinicaltrials.gov/ct2/show/NCT00912041. T11 is a 39 year-old man with tetraplegia due to a C4 spinal cord injury (ASIA Impairment Scale AIS-B) that occurred 11 years prior to enrollment in the trial. T5 was a 70 year-old man with tetraplegia due to a C4 spinal cord injury (AIS-C) that occurred 9 years prior to enrollment in the trial. At the time of this study, T11 had been enrolled in the trial for approximately 1 year, and T5 had been enrolled in the trial for approximately 6.5 years.

Permissions for this study were granted by the US Food and Drug Administration (FDA, Investigational Device Exemption #G090003) and the Institutional Review Boards (IRBs) of Massachusetts General Hospital (#2009P000505, initially approved 15 May, 2009, includes ceded review for Stanford University and Brown University) and Providence VA Medical Center (#2011-009, initially approved 9 March, 2011). This study was conducted in accordance with the Declaration of Helsinki. All research sessions were performed at the participants’ place of residence.

### 2.2. Intracortical recording and feature extraction

**Figure 1.**
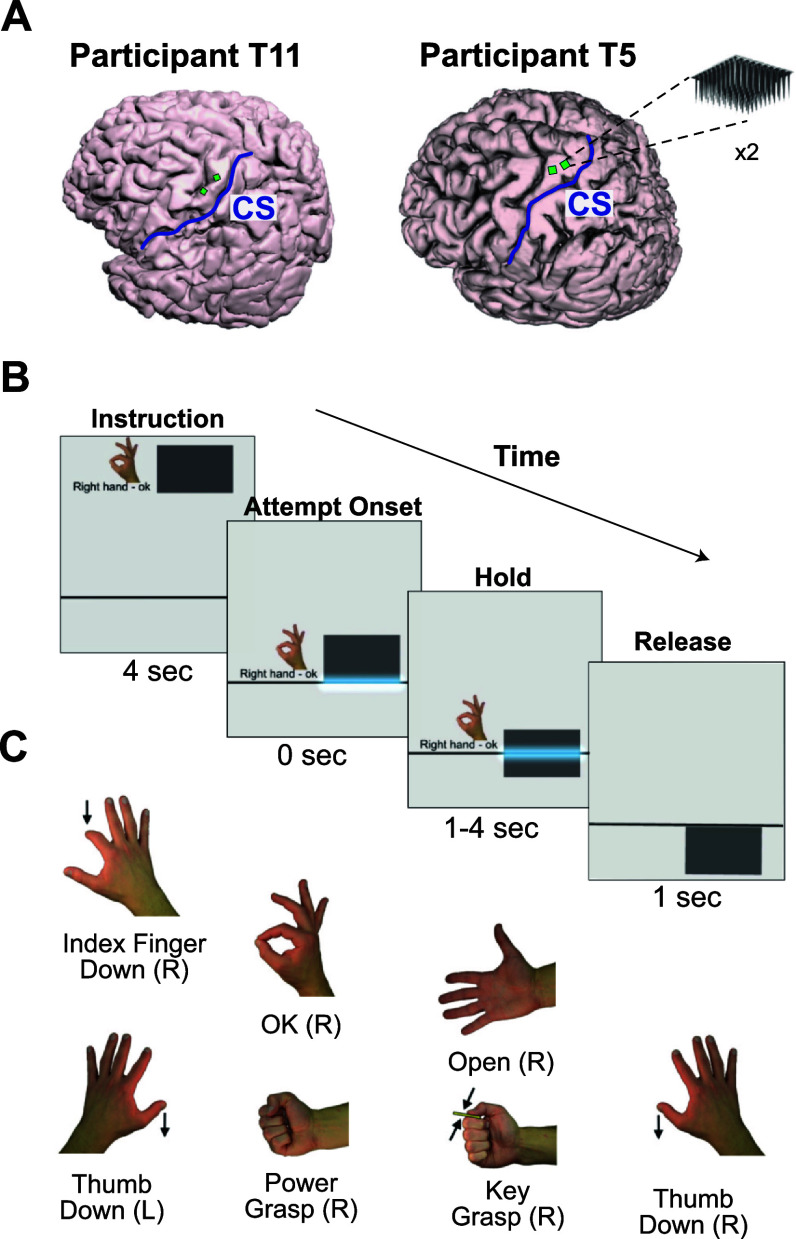
Recording locations and Gesture Hero task. (A) MRI-derived 3D reconstructions of the brains of participants T11 and T5 with the locations of implanted electrode arrays. Each participant had two 96-channel electrode arrays (green squares) implanted in the hand area of their left precentral gyrus. The central sulcus (CS) on each participant is denoted by a blue line. (B) Representative still frames from a Gesture Hero trial (see also video S1). Instruction: boxes with the upcoming gesture fell from the top of the screen to the attempt line (gray line) in 4 s. Attempt onset: the participant was requested to attempt and hold the gesture when the box contacted the attempt line. Hold: The hold period lasted for 1, 2, or 4 s. Release: after the box passed the attempt line, there was a 1.3 s intertrial interval before the next gesture box appeared. (C) Gestures presented in the task. Right hand: Index finger down, OK, Open, Key grasp, Power grasp, Thumb down. Left hand: Thumb down.

Each participant had two 96-channel microelectrode arrays (Blackrock Neurotech) placed chronically in the dorsal portion of the left precentral gyrus (PCG; figure 1(A)), which was determined via preoperative fMRI to represent the hand and arm area of motor cortex. Neural signals from T5’s electrode arrays were analog filtered (0.3 Hz to 7.5 kHz), digitally sampled at 30 kHz, and transmitted via two NeuroPort Patient Cables (Blackrock Neurotech). Neural signals from T11X were analog filtered (1.0 Hz to 7.8 kHz), digitally sampled at 20 kHz, transmitted wirelessly using two Brown Wireless Devices (Blackrock Neurotech), and then upsampled to 30 kHz sampling rate (via sample-and-hold) for compatibility with downstream processing. Previous work showed a negligible difference in signal quality between these two signal transmission strategies [21].

Using custom Simulink (Mathworks) software, we performed online feature extraction of seven neural features from each of the 192 signal channels in 20 ms time steps (figure S1). The 30 kHz raw neural signals were first decimated to 15 kHz and subjected to a common-average reference (CAR) [22]. Specifically, the mean of the 80 lowest-variance channels from each array, identified from a 1 min reference period at the start of the session, was subtracted from all channels on the same array.

Two spiking-related features, non-causal threshold crossings (TX) [23] and spike band power (SP), were extracted from each 20 ms segment. These segments were buffered with 4 ms of additional data (to prevent edge effects) and bandpass filtered using an 8th-order IIR Butterworth filter with cutoff frequencies of 250 Hz and 5000 Hz. For TX, the filtered signal was reversed in the time domain, bandpass filtered again, and threshold-crossing events were counted, where thresholds were set at −3.5 × the root mean square of the filtered signal voltage for each channel. SP was calculated as the total power of the signal after the initial bandpass filter, defined as $\sum({\mathrm{filtered data}})^2/300$, where 300 corresponds to the number of samples in the 20 ms window.

In addition to spiking features, five neural features representing the approximate power of local field potentials (LFPs) in specific frequency bands (0–11 Hz, 12–19 Hz, 20–38 Hz, 39–128 Hz, and 129–250 Hz) were calculated. After decimating the 15 kHz CAR-processed signal to 1 kHz, data were buffered to create 256-sample vectors representing the preceding 256 ms of sub-1 kHz neural activity. A fast-Fourier transform was applied, and the average power of frequency components within each frequency band was calculated.

This pipeline resulted in a 1344-dimensional feature vector, comprising seven features for each of the 192 channels, which was generated every 20 ms (see figure S1 for summary diagram). These feature vectors were either stored for offline analysis or used as inputs to online neural decoders, such as those employed during the Multi-Gesture Drag-and-Drop task (section 4).

## 3. Latch decoder development

### 3.1. Multi-duration gesture study: Gesture Hero task

To better understand how sustained gestures were represented in the neural recordings from our participants, we asked T11 and T5 to attempt hand gestures for differing durations using the Gesture Hero task [24]. Like its video game namesake (Guitar Hero, Activision) upcoming movements were instructed by falling boxes with a picture of the cued gesture (figure 1(B), video S1). The participants were asked to attempt and maintain the gesture when the falling box came in contact with the ‘attempt line’ positioned at the lower third of the screen and to relax after the box fell past the attempt line. Therefore, the attempt period was indicated by the amount of time the box intersected with the line. Since boxes fell at a constant speed, the size of the box was directly proportional to the attempt time, which was either 1, 2, or 4 s long. The instruction period, the time it took the box to move from the top of the screen to the attempt line, was 4 s. The intertrial period, the time between the box exiting the attempt line and the appearance of the next gesture box, was 1.3 s.

T11 was asked to attempt six right-handed gestures (index finger down, power grasp, OK, open, key grasp, thumb down) and one left-handed gesture (thumb down; figure 1(C)). He performed the Gesture Hero task on two session days, each session consisting of ten data blocks and twenty trials per gesture-duration condition. Due to time constraints, data collection with T5 was limited to using a subset of three gestures—index finger down, power grasp, and OK—in one session with twenty trials per gesture-duration condition.

### 3.2. Gesture information during sustained attempts

Using the Gesture Hero dataset, we investigated the representation of gesture information in our neural recordings over the course of three different hold durations. Specifically, we assessed *gesture selectivity*, defined as the proportion of neural features displaying significantly different values across at least two gesture conditions (Kruskal–Wallis (KW) test, *p* $ < $ 0.001), and *attempt selectivity*, defined as the proportion of features demonstrating significantly different values during gesture attempts compared to baseline (Wilcoxon Rank Sum (RS) test, *p* $ < $ 0.001). The KW and RS tests were applied on time-averaged windows of feature data (e.g. the mean of fifteen 20 ms time steps in a 300 ms window), thus the number of samples in each group represented the number of trials from each group (e.g. twenty 1 s power grip trials). We also used linear discriminant analysis (LDA) classification to evaluate basic decoder performance at different time points relative to the onset of gesture attempts.

**Figure 2.**
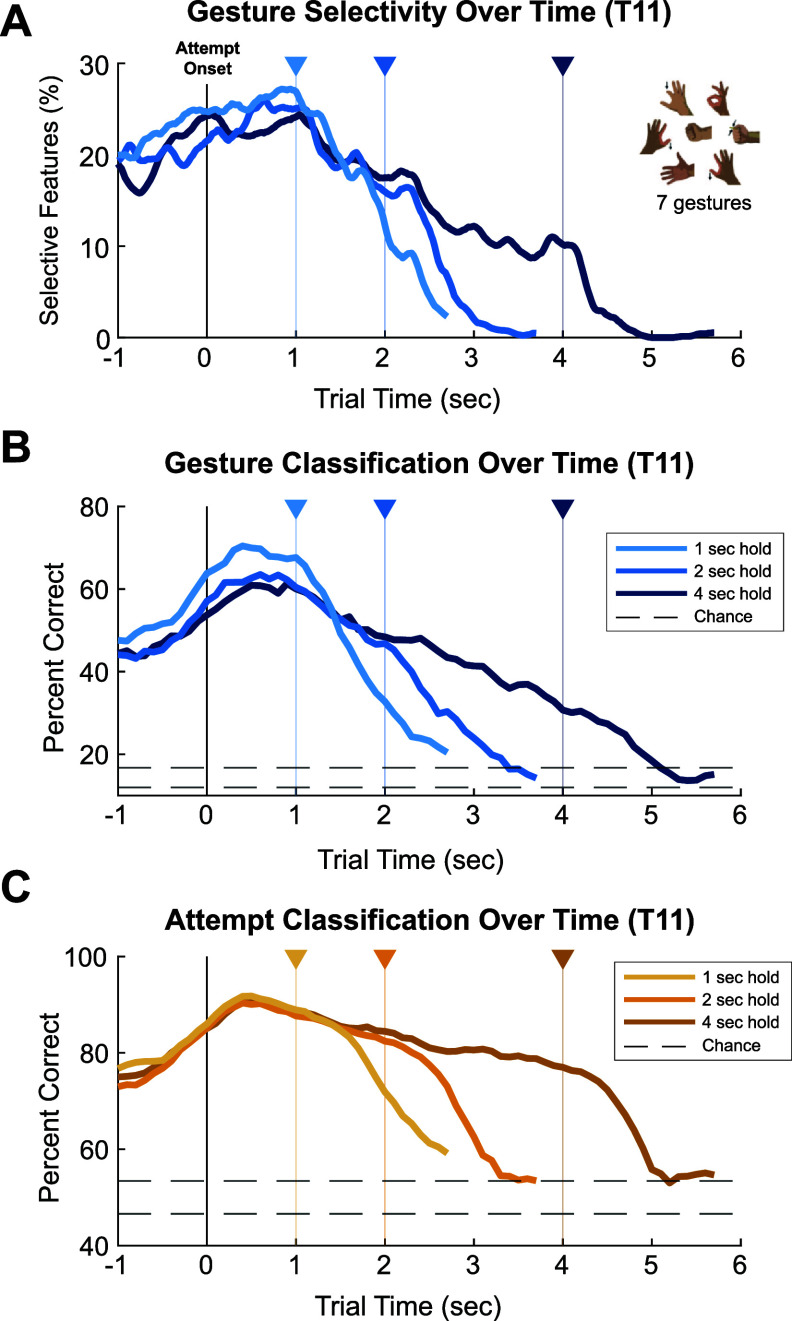
Gesture information in recordings from T11’s motor cortex during the Gesture Hero task. (A) The percent of neural spiking features (TX and SP) selective for gesture type assessed on 300 ms windows incremented in 20 ms increments over each set of trial durations (1 s, 2 s, and 4 s trials). Features were considered gesture selective in a given time window if they displayed significantly different mean values within the 300 ms window across the 7 gesture conditions (KW test, *p* $ < $ 0.001). Therefore, with 20 trials per gesture-duration condition, 20 mean TX or SP values comprised a group in the KW test. For T11, the number of gesture-selective features in each session were calculated independently and then averaged together. (B) The performance of an LDA classifier in decoding gestures across each trial duration. Cross-validated (5-fold) accuracies were computed on classifiers built on data from 500 ms windows stepped in 100 ms increments. Dashed lines reflect the 95% chance interval. (C) The performance of LDA classification in discriminating between all gestures and the intertrial period.

Similar to what was observed among neurons in NHPs [12, 13], gesture-selective activity appears to decrease during isometric holds of greater than 1 s (figure 2). For example, whereas about 25% of TX and SP features recorded from T11 were gesture selective after 1 s holds, 16% of features were gesture selective after 2 s holds, and 10% of features were gesture selective after 4 s holds (figure 2(A)). This diminishment in gesture selectivity was also present among LFP features (see figure S2(A)). Furthermore, by assessing the performance of an LDA classifier over the trial durations it becomes clear that standard LDA-based decoding approaches would not reliably maintain gesture decoding throughout extended attempt periods (figure 2(B), figure S2(B)). For example, during a 4 s sustained gesture attempt, a simple LDA classifier would correctly decode about 60% of 300 ms data samples centered at the 1 s mark after attempt onset, compared to 30% correct decodes at the 4 s mark, representing a 50% decrease in classifier performance. However, we found that if we applied LDA classification to the far simpler task of determining whether or not any gesture attempt was being performed (‘Attempt Classification’, figure 2C, S2C), we observed a less drastic drop off in decoding accuracy (11.5% decrease from 1 s to 4 s).

When applying the same analysis to the neural signals of T5 performing the Gesture Hero task (with only 3 gestures), we likewise found a significant drop off in gesture selectivity, with about 12% gesture-selective features at the end of 1 s hold trials, 6% at the end of 2 s hold trials, and 3% at the end of 4 s hold trials (figure S3(A)). Although gesture classification (LDA) performance assessed over time (figure S3(B)) also showed a decrease throughout T5’s sustained gesture attempts, this relative drop off (a 12.3% decrease from 1 s to 4 s) was far shallower than T11’s, even when compared with LDA classification of T11’s data evaluated on the subset of gesture trials performed by T5 (index finger down, power grasp, OK, 35.2% decrease from 1 s to 4 s; figure S4B). Curiously, attempt classification of T5 data did not demonstrate a meaningful decrease in accuracy over the trial duration (figure S3(C)), even revealing the presence of a second ‘peak’ shortly *after* the end of sustained gesture trials.

**Figure 3.**
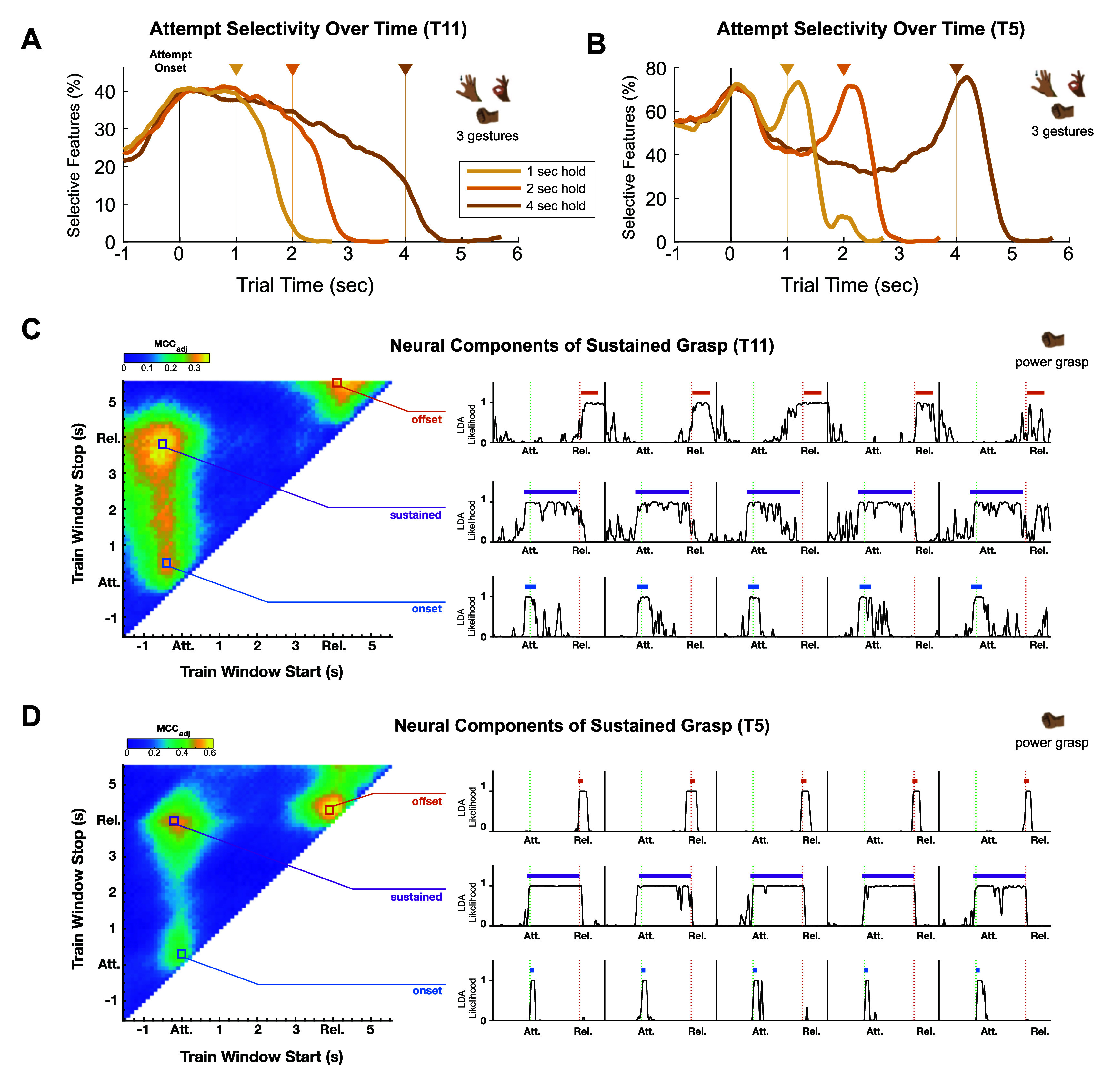
Comparison of neural components between participants. (A) and (B) The percent of neural features selective (TX and SP) for *attempt* assessed on 300 ms windows incremented in 20 ms increments over each set of Gesture Hero trial durations (1 s, 2 s, and 4 s trials). Features were considered attempt selective in a given time window if they displayed significantly different values within that window compared to baseline data (Wilcoxon Rank Sum *p* $ < $ 0.001). Baseline data were taken from the 300 ms window beginning 1.2 s after the end of different duration trials (e.g. attempt selectivity for 1 s trials was calculated by comparing the 1 s trial data to the baseline windows of the 2 s and 4 s trials.). Results shown using the subset of 3 gestures (index finger down, power grasp, and OK) for both T11 (A) and T5 (B). (C) and (D) Results of a grid search using only 4 s power grasp trials for T11 and T5 (see figure S6 for results from 1 s and 2 s trials). Each point on the heat plots on the left represent the performance (adjusted Matthews correlation coefficient; MCC$ _{\mathrm{adj}}$; 5-fold cross-validated) of a binary LDA classifier in differentiating between neural data within and outside of a given time window (‘Train Window’). The plots on the right display example LDA likelihood outputs from classifiers built on local maxima from the grid search process, described by Dekleva *et al* [20] as the ‘onset transient’ (blue), ‘offset transient’ (orange), and ‘sustained response’ (purple). Colored bars above the likelihood traces denote the start and end of the training window corresponding to each local maximum. The Attempt onset (‘Att.’) and Release (‘Rel.’; i.e. when the participant was supposed to start and stop attempting a power grip) are denoted by dotted green and red lines, respectively. Note that although there is a distinct ‘offset’ signal, centered at the Release mark, that emerges from the grid search on T5’s data (D), the corresponding local maximum identified in T11’s data is broadly centered over the intertrial period and more likely represents activity specific to the ‘relax’ state assumed by the participant between trials, rather than activity related to the release, or ‘offset’, of the power grip gesture.

We also assessed the percent of features that were attempt selective (i.e. combining data from all gesture types and comparing to baseline; RS test, *p* $ < $ 0.001) during Gesture Hero trials and found notable differences between T11 and T5 in the neural representations (figures 3(A), (B) and S5). Whereas, for T11, the percent of attempt-selective features peaked at the beginning of the trial and smoothly declined over time (figure 3(A)), for T5, attempt selectivity exhibited distinct peaks at both the onset and offset of the trial (figure 3(B)). Peaks at the beginning and end of T5 trials were most apparent in the neural spiking features, TX and SP, and in the highest frequency (129–250 Hz) LFP feature (figure S5(C)).

The presence of ‘onset’ and ‘offset’ responses in T5’s neural data resembles previous descriptions of distinct neural activity patterns, or *components*, associated with the onset, offset, and ‘sustained’ periods of single-gesture drag-and-drop trials performed by other iBCI participants [20]. These neural components were identified by performing an exhaustive grid search of all possible trial-aligned time windows, assessing the ability of a binary LDA classifier to differentiate between neural data collected within and outside of each time window [20]. When applying this approach to the 4 s Gesture Hero trials (TX and SP), we found peaks in classifier performance (measured by an ‘adjusted’ Matthews correlation coefficient (MCC$ _{\mathrm{adj}}$; see [20])) corresponding to onset and sustained components in both T11 and T5, but only data from T5 exhibited a distinct ‘offset’ component (figures 3(C), (D) and S6–S8).

The lack of reliable gesture offset components in T11 suggested that the approach of using two independent classifiers to control the onset and offset of sustained gesture attempts would not generalize well for all participants. We therefore designed a new approach that combines the transient, highly gesture-selective signal present at the beginning of a sustained gesture attempt (figures 2(A) and (B)) with the more robust, attempt-selective signal (figure 2(C)) present throughout the duration of a held gesture.

### 3.3. The Latch decoder

**Figure 4.**
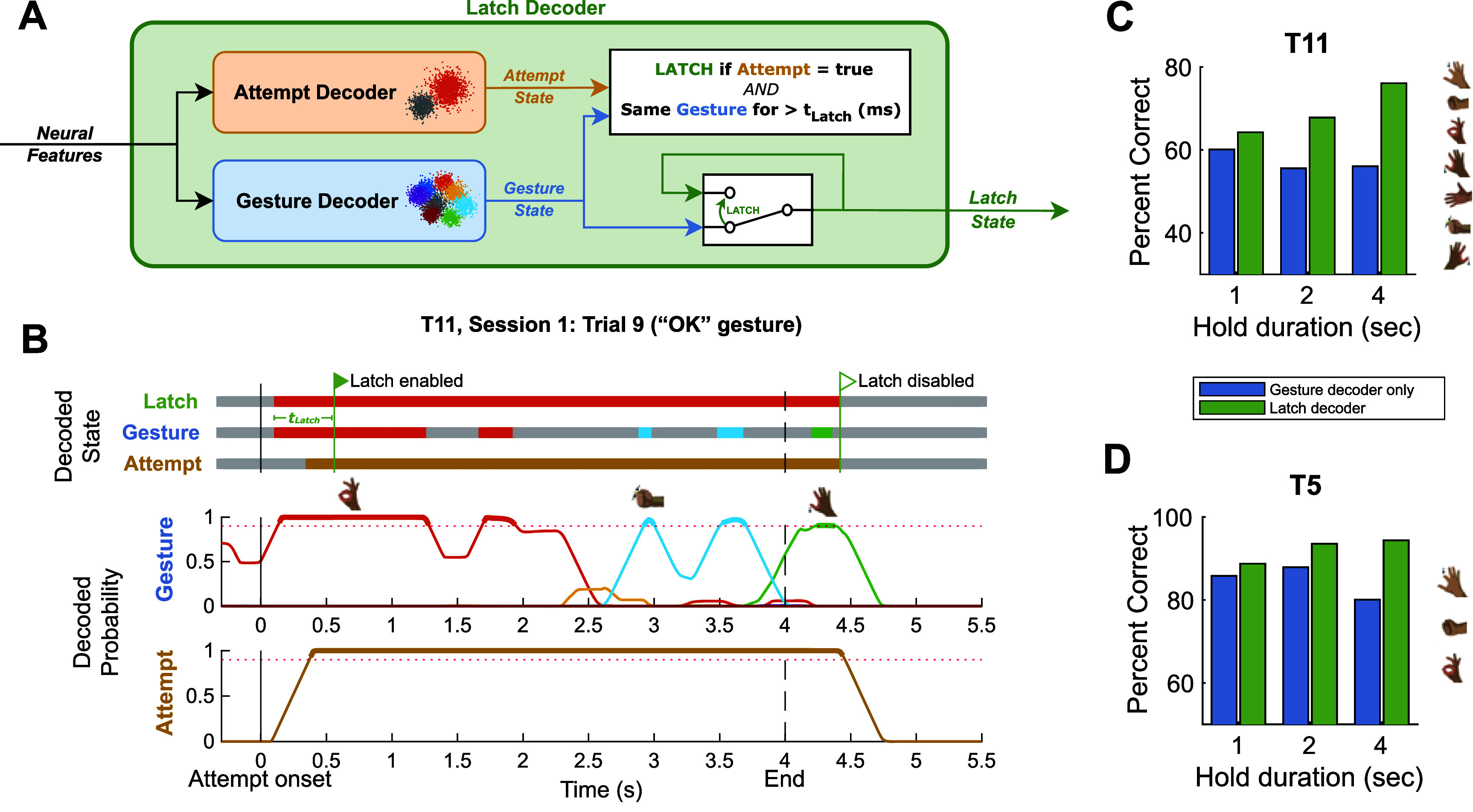
(A) Diagram detailing the Latch decoder pipeline. Neural features are input to both the binary Attempt decoder and the multi-class Gesture decoder, which run in parallel. If the Attempt decoder decodes an attempt and the Gesture decoder has decoded the same gesture type for more than 400 ms, then we ‘latch’ to the current decoded gesture type until the attempt decoder no longer decodes an attempt. (B) Decoded states (top) and normalized emission probabilities (bottom) from an example trial from the Gesture Hero task wherein T11 was prompted to hold an ‘OK’ gesture for 4 s. Around 200 ms after attempt onset, the Latch State and Gesture State both represent the correct gesture prediction. At $ _{\widetilde{~}}$600 ms, this initial gesture prediction is ‘latched’ because the Gesture State has been the same for 400 ms ($t_{\mathrm{Latch}}$) and the Attempt State is true. The Latch State returns to the relax/no-action state at $ _{\widetilde{~}}$4.5 s when the Attempt State becomes false. The Latch decoder compensates for multiple potential sources of error, including errors where the gesture is no longer decoded and when other gesture probabilities go above threshold during a hold period. (C) and (D) Offline cross-validated (10-fold) performance of the Gesture decoder (alone) and the Latch decoder on 1 s, 2 s, and 4 s Gesture Hero trials collected from T11 and T5. Bars represent the mean percent of correctly decoded time steps (20 ms steps) during the hold duration (i.e. for what percent of each hold period was the correct gesture decoded). To account for reaction time, only time steps after the first correctly decoded time step were evaluated.

Given that the peak in gesture-related information encoded in precentral neuronal activity was noted to occur at the onset of the attempted gesture, we developed a decoding strategy that would ‘latch’ or maintain the initial decoded gesture: the Latch decoder (figure 4(A)). The Latch decoder consists of two components. One component predicts the gesture type (Gesture), and the other predicts if any gesture is being attempted (Attempt). Initially, the Latch decoder’s output (Latch State) directly reflects the Gesture decoder’s state (Gesture State). However, when the Attempt State is true and the Gesture State has been the same for 400 ms ($t_{\mathrm{Latch}}$), the Latch State becomes *latched* to the present decoded gesture type until the Attempt State becomes false.

Figure 4(B) shows an example of how the Latch decoder operates. The Latch decoder’s state becomes latched $ _{\widetilde{~}}$600 ms after the attempt onset and does not change despite the changes in the Gesture decoder’s output. Its output becomes unlatched $ _{\widetilde{~}}$4.5 s into the trial when the Attempt State transitions to false. Using this approach on data collected from the Gesture Hero task, we found that the Latch decoder enabled substantial increases in the percent of correctly decoded time steps compared to using the Gesture decoder alone for both participants (figures 4(C) and (D)).

Both the Gesture and Attempt decoders used LDA in conjunction with a hidden Markov model (HMM), LDA-HMM, as specified in Hosman *et al* [26]. In brief, incoming *z*-scored features were smoothed with a 100 ms boxcar filter and projected to a low dimensional space before class posterior probabilities were computed using LDA. Emission probabilities were then produced via an HMM, smoothed with a 100 ms boxcar filter, and thresholded (see figure 4(B)) to determine the ‘decoded state’ output from each decoder.

Whereas the Gesture decoder was trained to differentiate between all gesture classes (including the relax state), the Attempt decoder was trained on the same data, but all gesture classes were relabeled as a single ‘attempt’ class. A regularization parameter (see [27]) empirically set to 0.3 was used when computing the LDA coefficients, and the class means and covariances used the empirical mean and covariances from the calibration data. For the Gesture decoder, the HMM transition matrix was set to match the gesture state transitions of the calibration task. The HMM transition matrix of the Attempt decoder was manually set to be extra ‘sticky’ [28], with on-diagonal values of $1-10^{-10}$, to prevent transient misclassification of the attempt signal.

**Figure 5.**
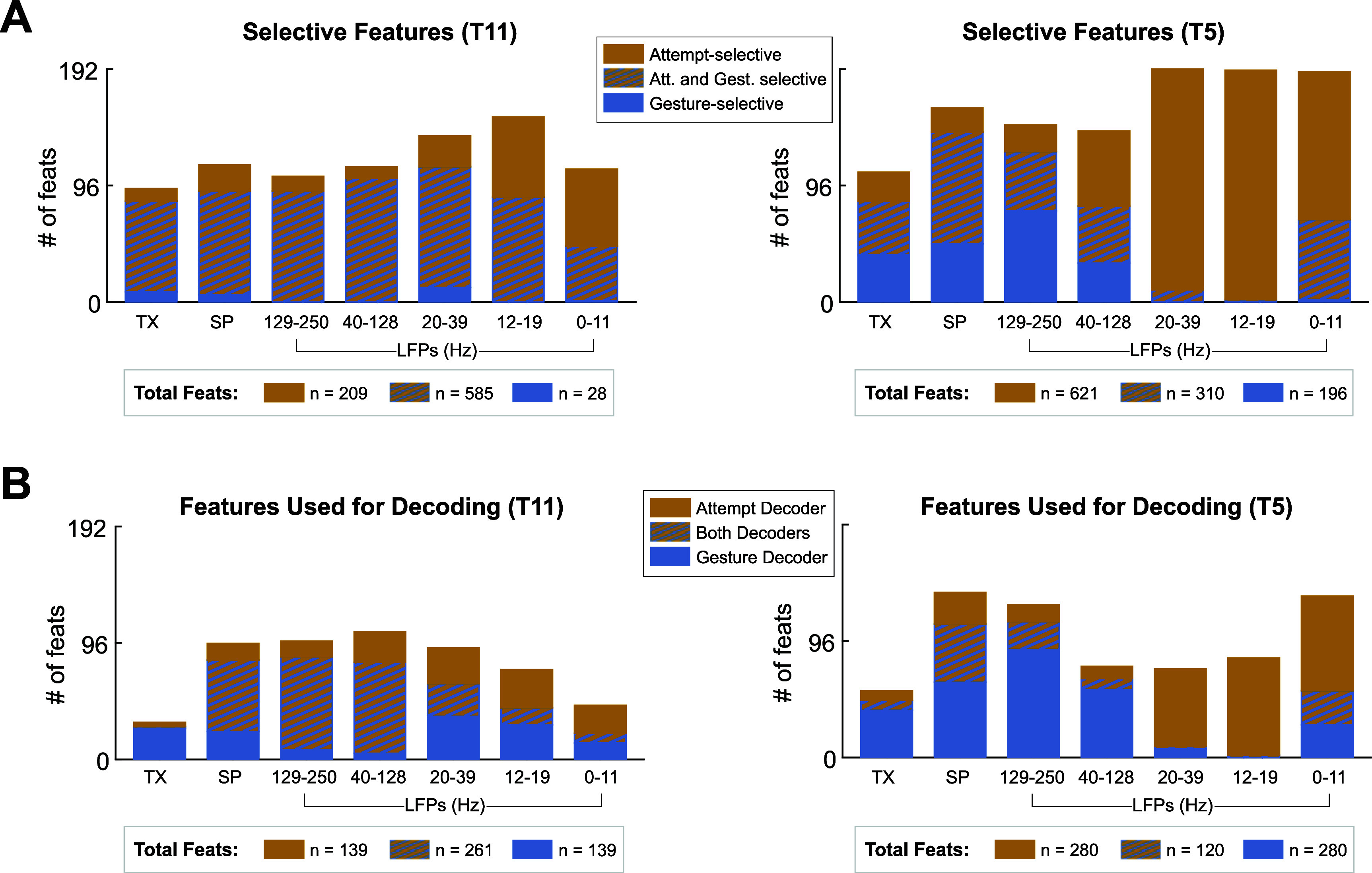
Feature selection for the Attempt and Gesture decoders. When building LDA-HMM decoders for attempt classification and gesture-type classification, an independent subset of 400 features are selected for each decoder by identifying all gesture (or attempt) selective features and choosing the top 400 features after ranking with a minimum redundancy maximum relevance (mRMR) algorithm [25]. (A) All gesture and attempt-selective features identified using data from all trials of the Gesture Hero task. Here, gesture-selective features were identified by performing a Kruskal–Wallis test (*p* $ < $ 0.001) on trial averages for each feature (7 groups/gestures for T11 and 3 groups/gestures for T5; 20 trials per gesture per session). Attempt-selective features were identified by performing a Wilcoxon Rank Sum test (*p* $ < $ 0.001), for each feature, on trial averages from all gesture trials compared to all intertrial period averages. (B) Each decoder was trained using the top 400 features according to the mRMR ranking. For the Gesture decoder, all gesture-selective features (from (A) were ranked using the fscmrmr(X,Y) function in MATLAB, where X represents the within-trial feature values (in 20 ms timesteps) for each feature and Y represents the gesture label at each timestep. For the Attempt decoder, all attempt-selective features were ranked similarly, but predictor data (Y) comprised both within-trial (labeled ‘attempt’) and inter-trial (labeled ‘no action’) periods. Analysis was performed on each session separately; plots for T11 represent the average of his two Gesture Hero sessions.

To ensure computational efficiency in our system, up to 400 of the 1344 recorded features were chosen for use by each decoder. First, all selective features were identified by performing a KW test (*p* $ < $ 0.001) on trial averaged data for each feature (figure 5(A)). Gesture-selective features were identified by comparing the within-trial averages (mean feature value between *attempt onset* and *release*) across all gesture conditions. Attempt-selective features were identified by comparing the within-trial averages to the inter-trial averages (mean feature value between *release* and *attempt onset* of the next trial). The top 400 gesture-selective features and top 400 attempt-selective features were then identified by ranking each set of features with the minimum redundancy maximum relevance (mRMR) algorithm [25] and selecting the 400 with the highest mRMR scores (figure 5(B)). Specifically, we used the fscmrmr(X,Y) function in MATLAB, where X represents the data from all gesture (or attempt) selective features at each 20 ms timestep, and Y represents the label at each time step. As before, the within-trial data (not trial averaged), labeled according to the specific gesture performed, were used in ranking the gesture-selective features, and the within-trial (labeled ‘attempt’) and inter-trial (labeled ‘no action’) data were used in ranking the attempt-selective features.

Although there is substantial overlap between features that are gesture-selective and attempt-selective (figure 5(A)), this feature selection process allows for the optimal selection of features for each decoding task. In particular, whereas the Gesture decoder utilized mostly TXs, SPs, and higher frequency LFP features, the Attempt decoder used very few TX features and used more LFP features (figure 5(B)).

## 4. Multi-gesture drag-and-drop study

Rarely are sustained gestures and clicks performed in isolation when using a personal computer or tablet. Thus, we designed a multi-gesture drag-and-drop task to assess how well the Latch decoder enables sustained grasp decoding *while* an iBCI participant is also controlling cursor kinematics.

### 4.1. Task description

**Figure 6.**
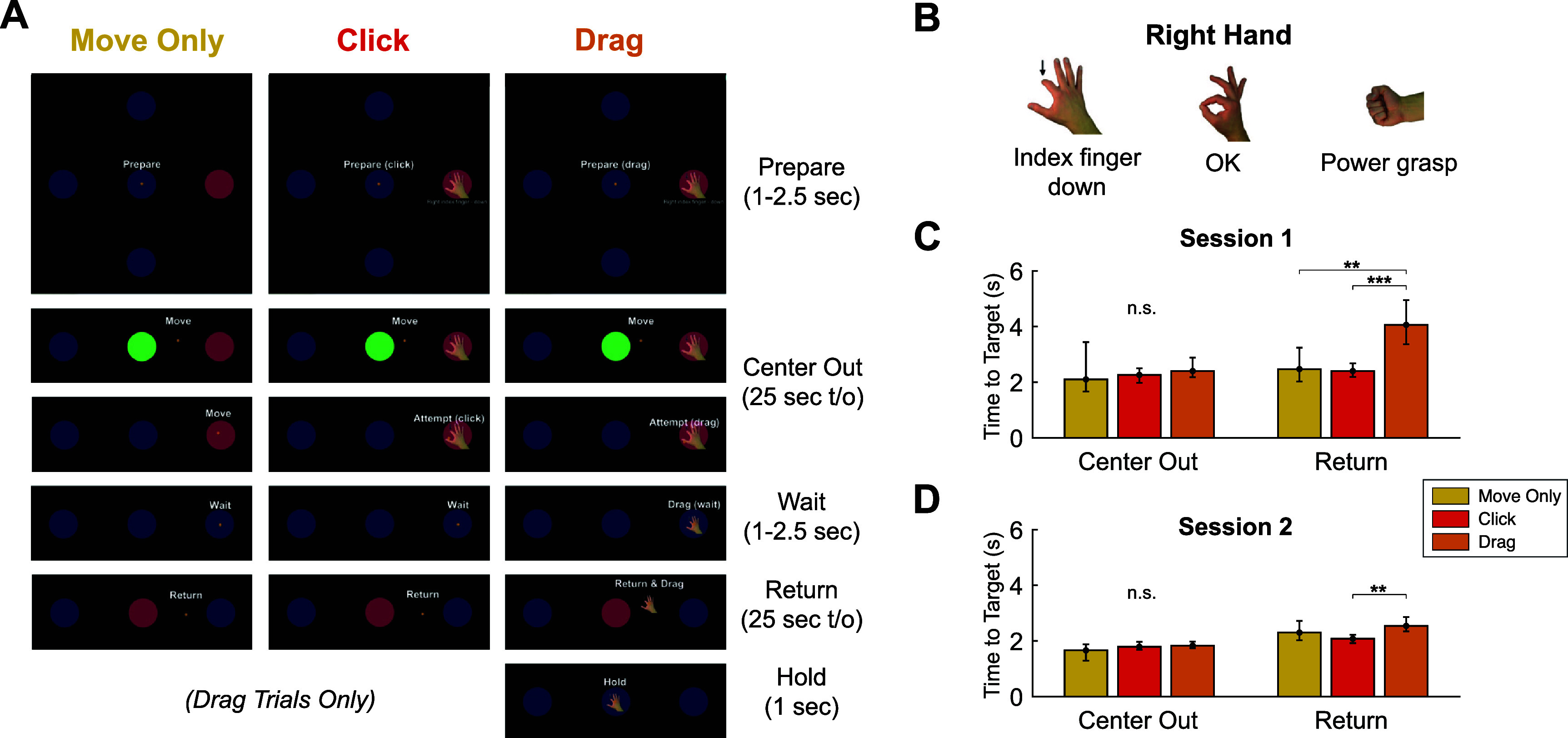
The Multi-Gesture Drag-and-Drop task. (A) Screenshots (cropped) from example trials where the participant acquired rightward targets under closed-loop two-dimensional iBCI control of a (small, orange) cursor. The task consisted of three trial variations: Move Only (no gesture decoding required), Click (transient gesture attempt needed to acquire outer target), and Drag (sustained gesture attempt required to acquire target and ‘drag’ the gesture icon to the center target). Each trial consisted of a combination of instructed delay stages (Prepare, Wait), self-timed stages with timeout (t/o) limits (Center Out, Return), and fixed duration stages (Hold; for Drag trials only). (B) Three gestures were used during Click trials and Drag trials of the Multi-Gesture Drag-and-Drop task. (C) and (D) Time to target results during the Center Out and Return stages for each trial variation in T11’s first session (C) and second session (D) performing the task. All attempts were limited to a 25 s timeout period. Bar heights represent the median time to target value for each trial variation. Error bars represent 95% confidence intervals. Horizontal bars with asterisks denote significant differences between trial types (Wilcoxon Rank Sum; *, *p* $ < $ 0.05; **, *p* $ < $ 0.01; ***, *p* $ < $ 0.001; n.s., *p* $ > $ 0.05).

The Multi-Gesture Drag-and-Drop task consisted of a 2D center out and return task with four outer targets positioned cardinally from the center target. There were three trial variations present in each data collection block (figure 6(A), video S2):

• **Move Only**: Move Only trials represented a simple kinematic task that required the participant to move a circular cursor from the center of the screen to an outer target (Center Out stage), wait for 1–2.5 s on the outer target (Wait), and move the cursor back to the center (Return). In order to acquire the outer target, the cursor needed to *dwell* within the target radius for 0.5 s.
• **Click**: During Click trials, the participant performed an identical kinematic task as for Move Only trials but was instructed to perform a transient gesture attempt, or ‘click’, in order to acquire the outer target.
• **Drag**: Drag trials were similar to Click trials, however, once the cursor reached the outer target, the participant was instructed to attempt and hold the cued gesture for 1–2.5 s and then continue holding the gesture while moving (i.e. dragging) the cued gesture icon from the outer target location back to the center. Drag trials contained an additional 1 s ‘Hold’ stage at the end wherein the participant continued attempting the gesture without kinematic movement.

Each trial began with a random 1–2.5 s ‘Prepare’ stage (figure 6(A)) wherein an outer target was cued by changing from blue to red. In Click trials and Drag trials the outer target was also overlaid with an image of one of the three gestures that they were supposed to perform once reaching the target (figure 6(B)). The words ‘Prepare (drag)’, ‘Prepare (click)’, or ‘Prepare’ were visible above the cursor during this stage. Thus, the participant had information on the movement direction, the gesture (if applicable), and the trial type he was about to perform from the beginning of the trial. To provide additional guidance during the task, the words ‘Move’, ‘Attempt’, ‘Drag’, ‘Return’, and ‘Hold’ were presented above the cursor (see figure 6(A)) to instruct the participant during each stage of the trial. During Drag trials, the gesture icon would shrink in size and follow the cursor to represent ‘dragging’ of the icon when the correct gesture was being decoded. If the decoder did not decode the correct gesture, the gesture icon would be ‘dropped’ (i.e. the icon would return to original size and cease moving along with the cursor). Both the Center Out and Return stages had timeouts of 25 s.

Participant T11 performed the Multi-Gesture Drag-and-Drop task during two sessions, each with a total of 11 blocks. Each block contained 4 Move Only trials (one for each direction), 12 Click trials (one for each direction/gesture combination), and 12 Drag trials (for each direction/gesture combination). The first four blocks were used to calibrate the decoders (steady state Kalman filter [29] for 2D kinematic decoding and the Latch decoder for gesture decoding) and the seven subsequent blocks were treated as assessment blocks.

All trials in the first block were ‘open loop’ (OL) trials, meaning the computer displayed idealized performance of the task while the participant imagined movements corresponding with the task. For this task, T11 imagined translating his right arm in a horizontal plane to create 2D movement of the cursor and performing the cued gestures with his right hand. For blocks 2-4, kinematic control was gradually given to the participant through step-wise reduction of error attenuation (EA). EA reduces the decoded cursor velocity in the error direction by a factor corresponding to how much assistance we want to provide to an incompletely calibrated kinematic decoder. For example, EA of 0.5 would reduce the decoded velocity component perpendicular to the cursor-target vector by half. Block 2, 3, and 4 had EA values of 0.5, 0.3, and 0.0, respectively, with new Kalman filters generated after each block that incorporated previous blocks’ data (see video S2). For all calibration blocks, gesture decoding was inactivated (i.e. remained completely OL). All seven assessment blocks were performed in ‘closed loop’ (CL), under full participant control (figure S9).

### 4.2. Kinematic performance

Within each trial of the Multi-Gesture Drag-and-Drop task there were two kinematic tasks: moving the cursor from the center to an outer target (Center Out stage) and returning from the outer target (Return). Despite contextual differences between Move Only, Click, and Drag trials, the instructed task during the Center Out stage was functionally the same between trial types. Thus, as expected, target acquisition times during this stage were not significantly different across trial types (KW, *p* = 0.265, figures 6(C) and (D)). However, trial timeouts were more common during Drag trials (2.4%) than Click trials (1.1%) and Move Only trials (0.0%).

During the Return stage, whereas Move Only and Click trials were functionally the same, Drag trials required the participant to perform the same kinematic task while simultaneously holding a gesture. T11’s median target acquisition times were significantly greater during the Drag trials (3.16 s) than for Click (2.22 s; Wilcoxon Rank Sum (RS), *p* = 3.92$\times 10^{-07}$), and Move Only (2.30 s; RS, *p* = 0.002) trials. However, this difference was far more noticeable during the first session (figure 6(C)) than the second session (figure 6(D)), suggesting a learning effect. Moreover, when considering Session 2 trials alone, Drag (Return) trial durations were not significantly different from Move Only trials (RS test, *p* = 0.186).

### 4.3. Latch decoder performance

During Drag trials of the Multi-Gesture Drag-and-Drop task, T11 used the Latch decoder to select and maintain selection of gesture icons across three trial stages: Wait, Return, and Hold. We evaluated the occurrence of gesture decoding errors during these epochs and compared performance to what would have occurred if the output of the Gesture decoder component was used on its own. Here, an error is considered an incorrect gesture decode, including a no gesture decode. Note that for the Wait period, to account for variance in reaction times, we only consider steps after the decode onset (correct or incorrect). If there was no decode during the Wait period, we counted the entire period as incorrect.

**Figure 7.**
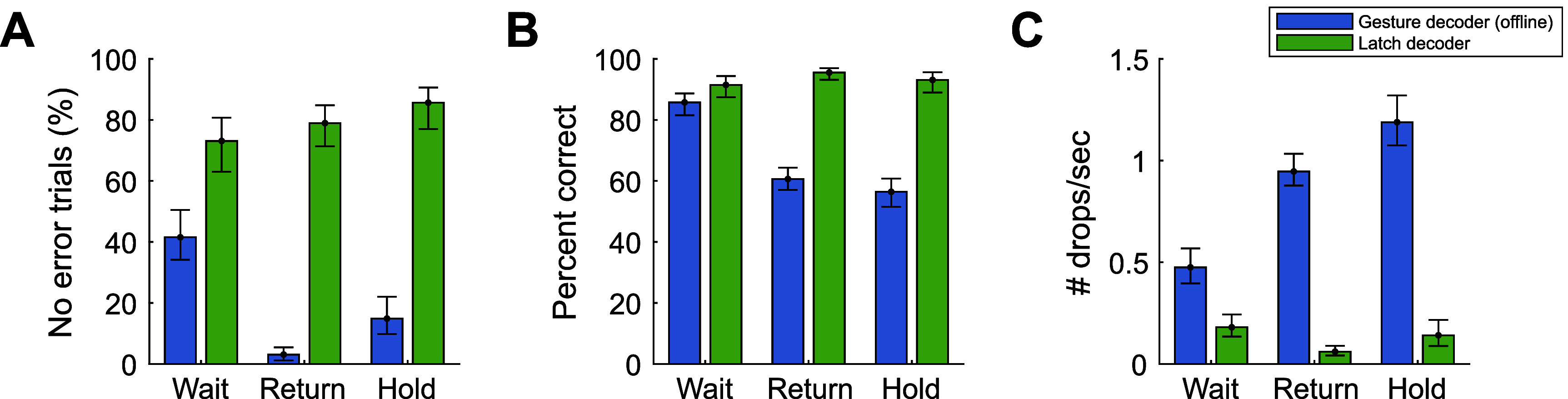
Performance of the Latch decoder compared to the Gesture decoder on its own (simulated offline) during Drag trials of the Multi-Gesture Drag-and-Drop task performed by T11. (A) The percent of trial epochs without any error. Estimated each block and showing means per trial epoch (Wait, Return, Hold) with 95% CI error bars. (B) Percent correctly decoded gesture across each individual trial. Mean across all trials with 95% CI error bars. (C) The number of dropped events per second. Mean across all trials with 95% CI error bars.

Using the Latch decoder, T11 completed 73% of Wait epochs, 79% of Return epochs and 86% of Hold epochs without a gesture decoding error. By contrast, if the output of the Gesture decoder was used on its own, only 41% of Wait epochs, 3% of Return epochs, and 15% of Hold Epochs would have been completed without error (figure 7(A)). Specifically, during Wait, Return, and Hold epochs, the Latch decoder, on average, output the correct gesture decode for 92%, 96%, and 93% of the duration of each epoch, respectively. Meanwhile, the Gesture decoder output reflected the correct gesture 86%, 61%, and 56% of the duration of Wait, Return, and Hold epochs, respectively (figure 7(B)).

The Latch decoder showed particular promise in preventing unintended drop events during the task, especially during the Return and Hold periods when the gesture attempt was being sustained beyond 1 or 2 s. Using the Latch decoder, T11 averaged 0.2, 0.1, and 0.1 drop events per second during the Wait, Return, and Hold periods, respectively. Using the Gesture decoder alone would have yielded 0.5, 0.9, and 1.2 drops per second, respectively.

The vast majority of drop events were due to errant ‘no action’ decodes. Of the 146 total drop events that occurred across all drag trials (Wait, Return, and Hold all included), 39 were associated with a subsequent decode of a non-target gesture, with a median decode duration of only 3 time steps (60 ms). There was no significant difference between the duration of non-target gesture decodes compared to the simulated results of using the Gesture decoder only (RS test, *p* = 0.24), as non-target gestures were only inadvertently ‘latched’ twice across all Drag trials.

The median gesture onset reaction time (i.e. time between when the cursor arrives on an outer target and when the correct gesture is first decoded) was 0.56 s. This was identical to the Gesture decoder results, because the Latch decoder, by definition, only differs from the Gesture decoder after a gesture is decoded for at least 400 ms. However, the gesture offset reaction time (calculated as the time between the end of the Hold stage and when the held gesture is no longer decoded) was significantly longer (RS test, *p* $ < < $ 0.001) for the Latch decoder (median of 0.84 s) compared to the Gesture decoder on its own (median of 0.11 s).

## 5. Discussion & conclusion

This study demonstrates that the Latch decoder can provide iBCI participants with significant improvements in the ability to ‘drag and drop’ icons using multiple gestures on a computer screen. The Latch decoder improves upon existing multi-gesture LDA-HMM classification of sustained gestures by taking advantage of the relatively higher neural discriminability of the overall ‘attempt’ signal to ‘latch’ to decoded gesture types inferred at the beginning of a movement period, capturing the phasic component of the neural response. Other approaches that were considered for accommodating decreasing signal quality during the hold period involved increasing the smoothing of neural features, increasing the time window on which the decoder was evaluated, or making the HMM transition matrix of the Gesture decoder more ‘sticky’ [28]. However, a drawback of these methods was that they introduced significant delays in the decoding of gesture onsets that would cause the experience of using the decoder to be unacceptably sluggish. A key benefit of the Latch decoder is that the accuracy and latency of decoding transient gesture attempts (or clicks) is unaffected because decoding of sustained gesture attempts is provided by a separate, parallel process that is only activated 400 ms after gesture onset.

Existing toggle-based approaches that use transient gesture attempts to drive changes in the controlled effector (e.g. hand flexion to close a robotic hand and hand extension to open the hand) require at least two distinct actions to control each degree of freedom. This can be seen as unintuitive for the user as the ‘return’ of many held postures can be more readily interpreted as a ‘release’ of a sustained motor attempt rather than activation of antagonistic muscle groups. Furthermore, this method potentially underutilizes 50% of the available motor repertoire, which could be used to drive additional degrees of freedom. Previous work describing the onset and offset of attempted gestures as distinct neural events in human motor cortex [20] presents a possibility that the initiation, maintenance, and release of an action like a mouse click could be controlled by a single gesture. However, like the toggle-based approaches, a decoder that classifies the onset and offsets of individual gestures requires two classifier states to control each gesture, and it is unclear how well this method might scale to decoding of multiple sustained gestures states.

The present study revealed a difference in the neural representations of gesture ‘offsets’ between participant T5 and T11, with a clear offset response recorded in T5 but not in T11 (figure 3, figure S6-S8). This difference could be attributed to slight differences in how each participant approached the task. Despite being given the same instructions, it is possible that T5 employed a more deliberate strategy to end gestures (e.g. envisioning opening the hand), while T11 took a more passive approach (e.g. simply relaxing when a gesture was released). Inter-participant differences in internal imagery are expected sources of variability in iBCI studies that ultimately can be influenced by difficult to control variables such as previous research activities performed using the iBCI or even prior experiences using computers before becoming paralyzed. Furthermore, despite T5 and T11 having arrays implanted in anatomically similar locations in the dorsal PCG (figure 1(A)), there is no guarantee that recorded signals represent activity from functionally comparable neural populations. Slight variations in array placement could have yielded a strong offset component in T11 and no offset in T5. In a prior study examining force decoding in motor cortex, a similar discrepancy emerged between T5, whose neural activity demonstrated onset and offset responses, and another participant with spinal cord injury, T8, whose neural activity more closely resembled T11’s when performing sustained grasp attempts [30]. Meanwhile, both participants in Dekleva *et al* [20] showed distinct onset and offset responses. It is important to note that the Latch decoder was designed to be effective for both participants with and without offset-related neural activation.

Given prior research on the phasic-tonic nature of motor cortical neurons [8, 9], the gradual decrease in gesture selectivity and classification performance over time among neural spiking features (figure 2), was not unexpected. Similar dynamics can be observed in previous iBCI studies where participants with tetraplegia were asked to perform sustained hand grasps [14, 30–32]. Our results lend support to the conclusion that motor cortical neurons primarily encode *changes* in intended muscle activation that, in an non-disabled individual, would affect changes in a motor state maintained by downstream spinal circuitry [12, 33]. The degradation of gesture discriminability over time appears to become most notable when more gestures are considered; gesture classification performance declined more rapidly when assessed on seven gestures (figure 2) vs. three gestures (figures S3 and S4). Therefore an approach like the Latch decoder, where the specific gesture type and the overall attempt signal are decoded through separate processes, will be increasingly useful as iBCIs are used to translate an increasing number of gestures.

With respect to iBCI decoding of arm reaches, LFPs have not been shown to contribute additional information compared to spiking activity, but can provide robustness when LFPs and spikes are used in combination [34–36]. When gesture classification of sustained gestures was performed on each of the feature sets on their own (figure S2(B)), we similarly found that spiking features (TX and SP) provided the best performance. However, there were many LFP features that were selective for gesture and attempt information (figure 5(A)), and even after optimization with the mRMR algorithm, which utilizes the mutual information metric to select maximally relevant features [25], many LFP features were prioritized over spiking features for inclusion in our decoders (figure 5(B)). An in-depth analysis of the representation of LFP features was not a focus of this study, although there is some evidence that LFPs can be informative for sustained gesture decoding, particularly for binary prediction of the attempt state (figures 5 and S5). It is also possible that because LFPs represent the summed activation of many neurons, they can offer more stable, albeit less rapidly responsive, signal sources compared to spiking features—a desirable property for an attempt decoder responsible for latching to gesture predictions without dropping. Further work is needed to determine the optimal utilization of these features.

The Gesture Hero task and Multi-Gesture Drag-and-Drop task were designed to evaluate sustained gestures performed for durations that would be expected during typical drag-and-drop control of items on a computer screen. There are other applications, for example the grasping and holding of a coffee cup with a robotic arm, that would demand stable sustained gesture control for much longer durations. Although T11’s neural activity displayed a more stable representation of attempt information compared to gesture information (figures 2, 3, S2, and S5), there was still a discernible drop off in attempt classification over time. Meanwhile, attempt classification for T5 (figure S3(C)) was remarkably stable. It is thus unclear how well the Latch decoder would support gestures sustained beyond 4 s, whether attempt information plateaus or extinguishes during longer holds, and how these dynamics differ across participants. Furthermore, the challenge of performing and maintaining a gesture for greater than, for example, 15 s could be mentally taxing for the user, at which point a toggle-based controller for decoding the onset and offset of gestures may become more desirable.

An important limitation of the Latch decoder is that the completion (or, offset) of a sustained gesture is necessarily governed by the dynamics of the Attempt decoder. In our implementation, the Attempt decoder is optimized to produce an accurate and reliable binary prediction of the attempt state with less concern for decoding latency because the onset of the action is decoded by the Gesture decoder. As a result, the Attempt decoder has greater smoothing, has a more ‘sticky’ transition matrix, and uses a greater number of low frequency LFP features than the Gesture decoder. A consequence is that releasing a latched gesture might feel delayed compared to releasing from a more momentary (less than 400 ms) gesture attempt. In the Multi-Gesture Drag-and-Drop task, trial offset time did not directly affect task performance, so it is unknown whether this slower gesture offset significantly affects user experience. Moreover, further work is needed to assess how well the Latch decoder accommodates more rapid user actions such as double-click or rapidly switching between gestures.

Preserving grasp-related decoding during translation is a recognized challenge [14]. Results from the Multi-Gesture Drag-and-Drop task indicate that the Latch decoder provides improved gesture decoding not only during isolated gesture holds, but also during simultaneous 2D kinematic control of the cursor (figures 7(A)–(C)). Compared to Click and Move Only trials, Drag trials took longer to complete, but this is also a behavior observed in drag-and-drop studies using standard (non-BCI) input methods [37] and could be due to cognitive factors unrelated to decoder performance [38]. Notably, T11’s performance of Drag trials greatly improved from Session 1 (figure 6(C)) to Session 2 (figure 6(D)), suggesting longer transport times could be overcome with practice. More work is needed to understand the neural representations of movement interactions during simultaneously controlled degrees of freedom during iBCI control.

The ability to hold specific gestures over longer time periods will expand the ways in which people using an iBCI can interact with screen-based devices like computers and tablets. Perhaps the most direct application of the Latch decoder will be on enabling more intuitive control of anthropomorphic robot arms or one’s own arm using functional electrical stimulation [18, 31]. However, further research will be needed to understand how well our approach translates to these other contexts.

## Data Availability

All data required to reproduce the findings in this study are publicly available on Dryad (https://doi.org/10.5061/dryad.98sf7m0v1). The dataset contains neural features (TX, SP, and LFPs) and task information collected during the two Gesture Hero sessions with T11, the one Gesture Hero session with T5, and the two Multi-Gesture Drag-and-Drop sessions with T11.

## Code availability

The code for reproducing the figures is made available at https://github.com/jgusman/drag-and-drop.

## References

[1] Hochberg L R, Serruya M D, Friehs G M, Mukand J A, Saleh M, Caplan A H, Branner A, Chen D, Penn R D, Donoghue J P (2006). Neuronal ensemble control of prosthetic devices by a human with tetraplegia. Nature.

[2] Collinger J L, Wodlinger B, Downey J E, Wang W, Tyler-Kabara E C, Weber D J, McMorland A J C, Velliste M, Boninger M L, Schwartz A B (2013). High-performance neuroprosthetic control by an individual with tetraplegia. Lancet.

[3] Gilja V (2012). A high-performance neural prosthesis enabled by control algorithm design. Nat. Neurosci..

[4] Andersen R A, Aflalo T, Kellis S (2019). From thought to action: the brain–machine interface in posterior parietal cortex. Natl Acad. Sci. USA.

[5] Guan C, Aflalo T, Kadlec K, Gámez de Leon J, Rosario E R, Bari A, Pouratian N, Andersen R A (2023). Decoding and geometry of ten finger movements in human posterior parietal cortex and motor cortex. J. Neural Eng..

[6] Klaes C (2015). Hand shape representations in the human posterior parietal cortex. J. Neurosci..

[7] Willett F R, Avansino D T, Hochberg L R, Henderson J M, Shenoy K V (2021). High-performance brain-to-text communication via handwriting. Nature.

[8] Smith A M, Hepp-Reymond M C, Wyss U R (1975). Relation of activity in precentral cortical neurons to force and rate of force change during isometric contractions of finger muscles. Exp. Brain Res..

[9] Maier M A, Bennett K M, Hepp-Reymond M C, Lemon R N (1993). Contribution of the monkey corticomotoneuronal system to the control of force in precision grip. J. Neurophysiol..

[10] Bennett K M, Lemon R N (1994). The influence of single monkey cortico-motoneuronal cells at different levels of activity in target muscles. J. Physiol..

[11] Baker S N, Spinks R, Jackson A, Lemon R N (2001). Synchronization in monkey motor cortex during a precision grip task. I. Task-dependent modulation in single-unit synchrony. J. Neurophysiol..

[12] Shalit U, Zinger N, Joshua M, Prut Y (2012). Descending systems translate transient cortical commands into a sustained muscle activation signal. Cereb. Cortex.

[13] Intveld R W, Dann B, Michaels J A, Scherberger H (2018). Neural coding of intended and executed grasp force in macaque areas AIP, F5 and M1. Sci. Rep..

[14] Downey J E, Weiss J M, Flesher S N, Thumser Z C, Marasco P D, Boninger M L, Gaunt R A, Collinger J L (2018). Implicit grasp force representation in human motor cortical recordings. Front. Neurosci..

[15] Kim S P, Simeral J D, Hochberg L R, Donoghue J P, Black M J (2008). Neural control of computer cursor velocity by decoding motor cortical spiking activity in humans with tetraplegia. J. Neural Eng..

[16] Kao J C, Nuyujukian P, Ryu S I, Shenoy K V (2017). A high-performance neural prosthesis incorporating discrete state selection with hidden Markov models. IEEE Trans. Biomed. Eng..

[17] Young D (2019). Closed-loop cortical control of virtual reach and posture using Cartesian and joint velocity commands. J. Neural Eng..

[18] Ajiboye A B (2017). Restoration of reaching and grasping movements through brain-controlled muscle stimulation in a person with tetraplegia: a proof-of-concept demonstration. Lancet.

[19] Colachis S C (2018). Dexterous control of seven functional hand movements using cortically-controlled transcutaneous muscle stimulation in a person with tetraplegia. Front. Neurosci..

[20] Dekleva B M, Weiss J M, Boninger M L, Collinger J L (2021). Generalizable cursor click decoding using grasp-related neural transients. J. Neural Eng..

[21] Simeral J D (2021). Home use of a percutaneous wireless intracortical brain-computer interface by individuals with tetraplegia. IEEE Trans. Biomed. Eng..

[22] Ludwig K A, Miriani R M, Langhals N B, Joseph M D, Anderson D J, Kipke D R (2009). Using a common average reference to improve cortical neuron recordings from microelectrode arrays. J. Neurophysiol..

[23] Masse N Y (2015). Reprint of non-causal spike filtering improves decoding of movement intention for intracortical BCIs. J. Neurosci. Methods.

[24] Vargas-Irwin C E (2024). Gesture encoding in human left precentral gyrus neuronal ensembles. bioRxiv.

[25] Ding C, Peng H (2005). Minimum redundancy feature selection from microarray gene expression data. J. Bioinform. Comput. Biol..

[26] Hosman T, Vargas-Irwin C E, Thengone D J, Kapitonava A, Hochberg L R, Simeral J D (2025).

[27] Friedman J H (1989). Regularized discriminant analysis. J. Am. Stat. Assoc..

[28] Fox E B, Sudderth E B, Jordan M I, Willsky A S (2008). An HDP-HMM for systems with state persistence.

[29] Brandman D M (2018). Rapid calibration of an intracortical brain–computer interface for people with tetraplegia. J. Neural Eng..

[30] Rastogi A (2021). The neural representation of force across grasp types in motor cortex of humans with tetraplegia. eNeuro.

[31] Bouton C E (2016). Restoring cortical control of functional movement in a human with quadriplegia. Nature.

[32] Wandelt S K, Kellis S, Bjånes D A, Pejsa K, Lee B, Liu C, Andersen R A (2022). Decoding grasp and speech signals from the cortical grasp circuit in a tetraplegic human. Neuron.

[33] Harel R, Asher I, Cohen O, Israel Z, Shalit U, Yanai Y, Zinger N, Prut Y (2008). Computation in spinal circuitry: lessons from behaving primates. Behav. Brain Res..

[34] Stavisky S D, Kao J C, Nuyujukian P, Ryu S I, Shenoy K V (2015). A high performing brain–machine interface driven by low-frequency local field potentials alone and together with spikes. J. Neural Eng..

[35] Bansal A K, Truccolo W, Vargas-Irwin C E, Donoghue J P (2012). Decoding 3D reach and grasp from hybrid signals in motor and premotor cortices: spikes, multiunit activity and local field potentials. J. Europhysiol..

[36] Hwang E J, Andersen R A (2013). The utility of multichannel local field potentials for brain–machine interfaces. J. Neural Eng..

[37] MacKenzie I S, Sellen A, Buxton W A S (1991). A comparison of input devices in element pointing and dragging tasks.

[38] Guillery E, Mouraux A, Thonnard J L (2013). Cognitive-motor interference while grasping, lifting and holding objects. PLoS One.

